# Role of Systemic Factors in the Progression to Vision‐Threatening Diabetic Retinopathy in a New Mexican Patient Population with Type 2 Diabetes: Glycemic Control Is Not Enough

**DOI:** 10.1155/jdr/7203748

**Published:** 2026-01-07

**Authors:** Rushi N. Mankad, Lauren Marek, Gabriella Acosta, Andrea P. Cabrera, Finny Monickaraj, Clifford Qualls, Arup Das

**Affiliations:** ^1^ Department of Ophthalmology & Visual Sciences, The University of New Mexico School of Medicine, Albuquerque, New Mexico, USA; ^2^ New Mexico Veterans Affairs Health Care System, Albuquerque, New Mexico, USA

**Keywords:** diabetes mellitus, diabetic macular edema (DME), diabetic retinopathy (DR), glycated hemoglobin (HbA1c), proliferative diabetic retinopathy (PDR)

## Abstract

**Purpose:**

The purpose of this study is to identify associative factors of vision threatening complications of diabetic retinopathy (DR), including proliferative diabetic retinopathy (PDR) and diabetic macular edema (DME) in a New Mexican patient population with Type 2 diabetes featuring a high proportion of Hispanic and American Indian patients.

**Methods:**

In a retrospective, case‐control, cross‐sectional study, we performed both univariate and multivariate logistic regression testing of systemic factors for association among patients with nonproliferative diabetic retinopathy (NPDR) without DME, NPDR with DME, and PDR. We also used receiver operating characteristic (ROC) curves to understand how reliable glycemic control is at predicting DR end‐complications.

**Results:**

Among our 584 patients, 172 were diagnosed with NPDR without DME, 293 with PDR, and 119 with NPDR with DME. A total of 25% of patients with NPDR without DME had poor glycemic control (HbA1c levels of ≥ 9.0%). Similarly, 10% of PDR and 7% of NPDR with DME patients had good glycemic control (HbA1c levels ≤ 7.0%), despite their advanced eye disease. For patients with PDR, we identified significant independent associations with microalbuminuria and macroalbuminuria, Hispanic ethnicity, duration of diabetes, and the use of insulin and beta‐blocker medications. In NPDR with DME patients, significant associations were noted with microalbuminuria and macroalbuminuria, HbA1c, GFR, and beta‐blocker medications. The hemoglobin A1c level was not significantly associated with PDR but was significantly associated with NPDR with DME. The ROC curve analysis indicated poor predictability for the HbA1c model concerning the presence of PDR (AUC = 0.640) and NPDR with DME (AUC = 0.640).

**Conclusions:**

This study suggests that glycemic exposure alone is not a good enough predictor of DR progression. Other diabetic microvascular complications such as nephropathy, in addition to race/ethnicity with differing genetic associative factors, may also play a role in the multifactorial progression through DR phenotypes.

## 1. Introduction

Many landmark clinical trials, such as the DCCT (Diabetes Control and Complications Trial), UKPDS (United Kingdom Prospective Diabetes Study), and ACCORD (Action to Control Cardiovascular Risk in Diabetes) Eye Study, have established the importance of strict blood glucose control in preventing or delaying the complications of diabetes mellitus, including diabetic retinopathy (DR) [1–3]. The severity of glycemia, as well as the duration of diabetes, hypertension, and hyperlipidemia, has repeatedly been shown to be the most decisive risk factors for developing and progressing through DR [4].

In the United States, Hispanics represent the largest ethnic minority group. Several studies have shown that the prevalence of diabetes is much higher (2–2.5 fold higher) in Hispanics of Mexican origin (22%–23.9%) compared to non‐Hispanic Whites [5]. Studies have also shown that Type 2 diabetes in these ethnic groups is associated with an increased severity of DR [6, 7]. New Mexico has the highest percentage of Hispanics or Latinos in the United States, 2.5 times higher than the national average of 19.4% [8]. In New Mexico, 48.6% of residents identify as Hispanic or Latino [8] and 12.4% identify as American Indians and Alaska Natives [9]. Since previous major trials have largely focused on these factors in non‐Hispanic Whites, we have a unique opportunity to assess these factors in this distinct setting. From a public health perspective, obtaining precise estimates of the prevalence and risk factors for DR in these ethnic groups is crucial. Our study is distinctive given our patient population, which encompasses ethnic and racial groups that display disproportionate levels of diabetes and retinopathy severity [6, 7, 10–12].

We aimed to identify associative factors for outcomes of sight‐threatening retinopathy like PDR or DME in a New Mexican cohort of patients with Type 2 diabetes. Also, we assessed whether a unidimensional HbA1c‐centered approach is adequate at predicting retinopathy severity in this population.

## 2. Methods

This cross‐sectional retrospective study was designed to determine the factors associated with vision‐threatening DR in patients confirmed to have Type 2 diabetes between February 1, 2018 and February 1, 2020. The study protocol was approved by the University of New Mexico Institutional Review Board (UNM HRRC Study 16‐447) before the start of this study.

### 2.1. Subjects

Our study cohort consisted of 584 patients with diabetes who were examined at the University of New Mexico Health Sciences Center (UNMHSC), Albuquerque Eye Clinic. Data were collected between February 15, 2020 and June 15, 2021. Retrospective patient data were obtained via the UNMHSC′s Cerner PowerChart patient charting system. International Classification of Diseases, 10^th^ edition (ICD‐10) codes were used to generate patient lists with PDR, NPDR with DME, or NPDR without DME diagnoses.

### 2.2. Systemic Factors

The studied factors were selected after a literature review of studies that determined mixed conclusions about their associations with developing PDR or DME. These potential factors were collected using standardized methods at subjects’ regularly scheduled appointments with any UNMHSC healthcare provider.

Age, sex, and race/ethnicity were collected from patient demographic information self‐reported by patients at their initial visits; patients′ races/ethnicities were sorted into either non‐Hispanic Whites, Hispanics, American Indians, or others. HbA1c values were collected from February 2010 to February 2020 and averaged to calculate a 10‐year mean value. Duration of diabetes was determined from patient‐reported diagnosis year. During the last visit, insulin, angiotensin converting enzyme (ACE) inhibitor, beta blocker, and calcium channel blocker medications were chosen from patients′ active medication lists. Body mass index (BMI) in kilograms per square meter was calculated using patients′ last height and weight. Systolic blood pressure (SBP) and diastolic blood pressure (DBP) in millimeters of mercury, total cholesterol, low density lipoprotein (LDL), high density lipoprotein (HDL), and triglyceride levels in grams per deciliter were obtained from February 2018 to February 2020 and averaged to calculate 2‐year mean values. The glomerular filtration rate (GFR) in milliliters per minute per 1.73 m^2^ was calculated using the Chronic Kidney Disease Epidemiology Collaboration Equation [13], and the last reported value was used. Albuminuria was assessed using the patient′s latest urine albumin‐to‐creatinine ratio (UACR) in milligrams of albumin per gram of creatinine and then qualified as either normoalbuminuria (≤ 30 mg/g), microalbuminuria (30 < UACR ≤ 300 mg/g), or macroalbuminuria (> 300 mg/g) as per the National Kidney Foundations′ set values. History of dialysis, smoking, myocardial infarction, stroke, and cancer were determined using review of patients′ past medical and social history.

### 2.3. Retinopathy Assessment

Each case was reviewed to confirm that ICD‐10 coded diagnoses matched the actual phenotype. Ocular exams were performed on all patients, including best corrected visual acuity, stereoscopic biomicroscopy, and indirect ophthalmoscopy. Fundus photographs (wide angle OPTOS) were obtained in 74% of subjects and optical coherence tomography images (Heidelberg Spectralis) were done in 100% of subjects. The phenotype of each case was confirmed by the senior retina specialist (A.D.) based on clinic notes and examination drawings, fundus photographs, and OCT images. Cases were excluded if their ICD‐10 coding did not match phenotypic confirmation on chart review or if there was any other discrepancy.

### 2.4. Outcomes

Three cohorts of patients with diabetes were included in this study. (i) NPDR without DME was confirmed in cases which displayed only a few microaneurysms without any macular edema and a duration of diabetes of at least 10 years. (ii) PDR cases were confirmed by the presence of neovascularization in the retina or evidence of panretinal photocoagulation laser (PRP) and/or pars plana vitrectomy (PPV) surgery. (iii) NPDR with DME was confirmed in cases that exhibited hard exudates or retinal thickening per optical coherence tomography with central retinal thickening greater than 320 *μ*m in males and 305 *μ*m in females [14].

### 2.5. Statistical Analysis

Outcomes of either PDR vs. NPDR without DME, or NPDR with DME vs. NPDR without DME were analyzed. Both univariate and multivariate logistic regression analyses were done to study the effect of various associative factors. Baseline means and proportions were compared using two sample Student′s *t* tests or chi‐squared tests separately for the PDR or DME outcomes. The multivariate regression models for the PDR or DME outcomes were obtained using backward stepwise multivariate logistic regression and verified by the forward stepwise procedure. Candidate variables for the multivariate procedures for each outcome were the associative factors listed in “Systemic Factors” above that were statistically significant in univariate logistic regressions. Univariate and multivariate results were reported using odds ratios, 95% confidence intervals, and *p* values.

To compare the multivariate model with the univariate HbA1c model, receiver operating characteristic (ROC) curves and areas under the curve (AUC) were computed. The clinical interpretation of AUC used the following scale: the minimum AUC of 0.5 describes a test as no better at predicting disease vs. nondisease than by chance, whereas the maximum AUC of 1 means that the test is perfect in its differentiation [15], AUC of 0.7–0.8 is considered acceptable, 0.8–0.9 excellent, and greater than 0.9 is considered outstanding. *p* Values ≤ 0.05 were considered significant. All statistical analyses were conducted with SAS software, Version 9.4.

## 3. Results

Of our 584 subjects, three cohorts were confirmed to have: (i) NPDR without DME (172), (ii) PDR (293), and (iii) NPDR with DME (119). Combining all cohorts together who disclosed their racial identity, the Hispanics constituted 24.0% of total patients, and the American Indians constituted 10.1% of the total number of patients.

As per univariate logistic regression, PDR was significantly associated with younger age (*p* < 0.001), higher HbA1c (*p* < 0.001), duration of diabetes (*p* < 0.001), higher SBP (*p* < 0.001), lower GFR (*p* < 0.001), Hispanic ethnicity (< 0.001), microalbuminuria (*p* < 0.001) and macroalbuminuria (p 0.023), use of insulin medication (*p* < 0.001), use of beta‐blocker medication (*p* < 0.001), history of dialysis (*p* < 0.001), history of smoking (*p* < 0.001) and stroke (p <0.01) (Table 1) and, NPDR with DME was significantly associated with younger age (*p* < 0.001), higher SBP (p <0.006), lower GFR (p <0.014), microalbuminuria (*p* < 0.001), macroalbuminuria (*p* < 0.001), and use of beta blocker (*p* < 0.001) (Table 1).

**Table 1:** Univariate association with proliferative diabetic retinopathy (PDR) and NPDR with diabetic macular edema (DME).

	**NPDR without DME (** **n** = 172**)**	**PDR (** **n** = 293**)**			**NPDR with DME (** **n** = 119**)**		
**Variable**	**Mean (SD)**	**Mean (SD)**	**OR (95% CI)**	**p**	**Mean (SD)**	**OR (95% CI)**	**p**
Age (years)	68.3 (11.2)	60.5 (11.0)	0.937 (0.919, 0.956)	< 0.001	64.3 (10.7)	0.968 (0.947, 0.990)	0.004
HbA_1c_ (%)	8.2 (1.4)	9.0 (1.8)	1.340 (1.167, 1.540)	< 0.001	9.0 (1.7)	1.349 (1.134, 1.604)	< 0.001
Duration of DM (years)	20.0 (0.8)	20.4 (1.0)	1.927 (1.370, 2.709)	< 0.001	17.0 (0.9)	0.963 (0.925, 1.004)	0.075
BMI (kg/m^2^)	30.5 (6.3)	30.8 (6.8)	1.009 (0.978, 1.041)	0.591	30.6 (7.0)	1.002 (0.965, 1.042)	0.899
SBP (mmHg)	132.5 (15.1)	139.0 (16.5)	1.027 (1.012, 1.042)	< 0.001	138.5 (17.3)	1.024 (1.007, 1.041)	0.006
DBP (mmHg)	69.9 (7.1)	71.4 (8.2)	1.025 (0.997, 1.055)	0.081	71.7 (7.5)	1.034 (0.998, 1.072)	0.066
Total cholesterol (g/dL)	154.3 (37.6)	155.7 (47.5)	1.001 (0.995, 1.006)	0.783	165.4 (68.2)	1.004 (0.999, 1.010)	0.126
LDL (g/dL)	75.2 (30.3)	79.0 (36.3)	1.003 (0.996, 1.011)	0.340	85.1 (52.7)	1.006 (0.999, 1.013)	0.082
HDL (g/dL)	46.7 (14.8)	49.4 (36.4)	1.004 (0.994, 1.014)	0.443	47.7 (14.2)	1.005 (0.986, 1.024)	0.609
Triglycerides (g/dL)	176.7 (124.1)	164.6 (122.9)	0.999 (0.997, 1.001)	0.420	173.9 (127.3)	1.000 (0.998, 1.002)	0.870
GFR (mL/min/1.73m^2^)	63.1 (26.8)	49.3 (30.0)	0.984 (0.976, 0.991)	< 0.001	53.9 (31.1)	0.989 (0.980, 0.998)	0.014
Sex (female)	92 (56.1)	144 (52.2)	0.854 (0.579, 1.259)	0.423	55 (46.2)	0.673 (0.419, 1.081)	0.101
Race/ethnicity				< 0.001			< 0.001
White	100 (69.4)	75 (35.1)	Reference	—	52 (60.5)	Reference	—
American Indian	10 (6.9)	31 (14.5)	4.133 (1.908, 8.955)	0.053	4 (4.7)	0.769 (0.230, 2.572)	0.44
Hispanic	21 (14.6)	97 (45.3)	6.158 (3.522, 10.768)	< 0.001	24 (27.9)	2.198 (1.119, 4.315)	0.02
Other	13 (9.0)	11 (5.1)	1.128 (0.479, 2.658)	0.029	6 (7.0)	0.888 (0.319, 2.471)	0.59
Albuminuria				< 0.001			< 0.001
Normoalbuminuria	57 (50.9)	14 (10.1)	Reference	—	1 (1.5)	Reference	—
Microalbuminuria	35 (31.3)	91 (65.5)	6.921 (3.097, 15.469)	< 0.001	41 (60.3)	66.771 (8.788, 507.33)	< 0.001
Macroalbuminuria	20 (17.9)	34 (24.5)	10.585 (5.243, 21.374)	0.023	26 (38.2)	74.100 (9.433, 582.10)	< 0.001
Use of insulin	90 (54.6)	213 (78.3)	3.008 (1.975, 4.582)	< 0.001	77 (64.7)	1.528 (0.941, 2.481)	0.087
Use of ACE‐inhibitor	73 (46.8)	115 (43.6)	0.878 (0.590, 1.306)	0.520	45 (40.9)	0.787 (0.481, 1.289)	0.342
Use of calcium channel blocker	47 (30.3)	76 (28.4)	0.910 (0.590, 1.403)	0.668	39 (36.1)	1.299 (0.771, 2.187)	0.325
Use of beta blocker	54 (34.8)	40 (14.9)	0.328 (0.205, 0.526)	< 0.001	16 (14.8)	0.325 (0.174, 0.608)	< 0.001
History of dialysis	10 (6.6)	53 (22.3)	4.068 (1.999, 8.275)	< 0.001	14 (13.1)	2.138 (0.911, 5.014)	0.081
History of smoking	68 (44.2)	67 (26.7)	0.461 (0.302, 0.703)	< 0.001	36 (33.6)	0.641 (0.384, 1.070)	0.089
History of myocardial infarction	11 (7.1)	7 (2.7)	0.358 (0.136, 0.945)	0.038	2 (1.8)	0.245 (0.053, 1.130)	0.071
History of stroke	16 (10.4)	10 (3.8)	0.344 (0.152, 0.778)	0.010	9 (8.3)	0.784 (0.333, 1.846)	0.578
History of cancer	16 (10.5)	16 (7.5)	0.697 (0.344, 1.413)	0.317	18 (16.2)	1.657 (0.804, 3.415)	0.171

*Note:* Baseline means and proportions of study subjects with NPDR without DME, PDR, or NPDR with DME. Univariate analysis of each variable comparing either PDR vs. NPDR without DME or NPDR with DME vs. NPDR without DME reported with odds ratios, 95% confidence intervals, and *p* values.

**Figure 1:**
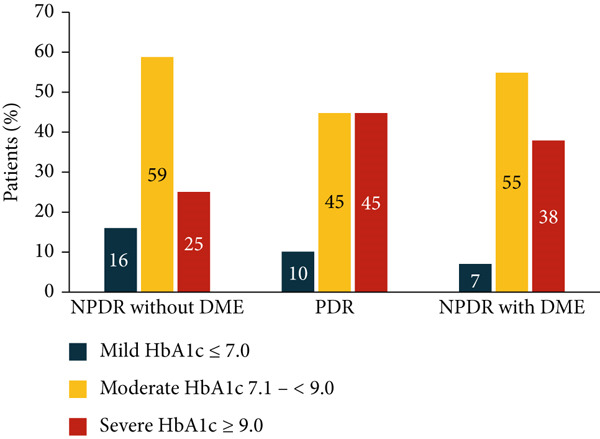
Classification of study subjects with NPDR without DME (left), PDR (center), or NPDR with DME (right) by HbA1c severity: Mild HbA1c ≤ 7.0 (blue), moderate 7.0 < HbA1c < 9.0 (yellow), and severe HbA1c ≥ 9.0 (red).

The majority (75%) of patients with NPDR without DME had good or moderate glycemic control (Figure 1). Interestingly, 25% of NPDR without DME patients had poor glycemic control (HbA1c > 9.0). Further, most patients with PDR (55%) and DME (62%) had good (HbA1c < 7.0) or moderate glycemic control (HbA1c 7.1–< 9.0).

Associative factors independently associated with PDR development as per multivariate analysis included albuminuria severity (microalbuminuria OR = 36.255 vs. normoalbuminuria [CI 7.223, 181.983] and macroalbuminuria OR = 36.958 vs. normoalbuminuria [CI 6.553, 208.440], *p* < 0.001), race/ethnicity (Hispanic ethnicity OR = 7.594 vs. non‐Hispanic White [CI 2.195, 26.272], *p* = 0.012), duration of diabetes (OR = 1.828 per additional year with diagnosis [CI 1.076, 3.104], *p* = 0.026), and use of insulin (OR = 4.895 vs. no use [CI 1.430, 16.758], *p* = 0.011); use of beta‐blocker medication (OR = 0.157 vs. no use [CI 0.049, 0.508], *p* = 0.002) was shown to be inversely associated (Table 2). However, the HbA1c level did not significantly correlate with the prevalence of PDR.

**Table 2:** Multivariate model of PDR vs. NPDR without DME and NPDR with DME vs. NPDR without DME.

**PDR vs. NPDR without DME**			**NPDR with DME vs. NPDR without DME**		
**Variable**	**OR (CI)**	**p**	**Variable**	**OR (CI)**	**p**
Albuminuria		< 0.001	Albuminuria		< 0.001
Normoalbuminuria	Reference		Normoalbuminuria	Reference	
Microalbuminuria	36.255 (7.223, 181.983)		Microalbuminuria	62.187 (8.028, 481.687)	
Macroalbuminuria	36.958 (6.553, 208.440)		Macroalbuminuria	47.271 (5.656, 395.105)	
Race/ethnicity		0.012	HbA_1c_ (%)	1.353 (1.025, 1.785)	0.033
White	Reference		GFR (mL/min/1.73 m^2^)	0.984 (0.970, 0.998)	0.027
Hispanic	7.594 (2.195, 26.272)		Use of beta blocker	0.219 (0.088, 0.544)	0.001
Duration of DM (years)	1.828 (1.076, 3.104)	0.026			
Use of insulin	4.895 (1.430, 16.758)	0.011			
Use of beta blocker	0.157 (0.049, 0.508)	0.002			

*Note:* Multivariate analysis results comparing either PDR vs. NPDR without DME (left) or NPDR with DME vs. NPDR without DME (right) reported with odds ratios, 95% confidence intervals, and *p* values. Only significant variables are listed.

In the model for NPDR with DME, significant associative factors included albuminuria severity (microalbuminuria OR = 62.187 vs. normoalbuminuria [CI 8.028, 481.687] and macroalbuminuria OR = 47.271 vs. normoalbuminuria [CI 5.656, 395.105], *p* < 0.001) and HbA1c (OR = 1.353 per additional percent [CI 1.025, 1.785], *p* = 0.033); both GFR (OR = 0.984 per additional mL/min/1.73m^2^ [CI 0.970, 0.998], *p* = 0.027) and use of beta‐blocker medication (OR = 0.219 vs. no use [CI 0.088, 0.544], *p* = 0.001) were shown to be inversely associated (Table 2). In contrast to our findings in the PDR cohort, HbA1c levels were significantly associated with the presence of DME.

The univariate HbA1c model was “poor” at predicting disease severity for both PDR (AUC = 0.640) and NPDR with DME (AUC = 0.640) from NPDR without DME (Figure 2). In contrast, our multivariate models were “excellent” predictors for development of PDR (AUC = 0.895) and NPDR with DME (AUC = 0.850) from NPDR without DME.

Figure 2Receiver operating characteristic (ROC) curves with calculated area under the curve (AUC) statistics. The univariate HbA1c model (red) is compared with the multivariate model (blue) at predicting disease severity. The models are tested at how well they predict PDR vs. NPDR without DME (a) and predicting NP vs. NPDR without DME (b). The multivariate model is “excellent” at predicting both PDR (AUC = 0.895) and DME (AUC = 0.850) vs. NPDR without DME. The univariate HbA1c model is “poor” at predicting both PDR (AUC = 0.640) and DME (AUC = 0.640) vs. NPDR without DME.

**Figure:**
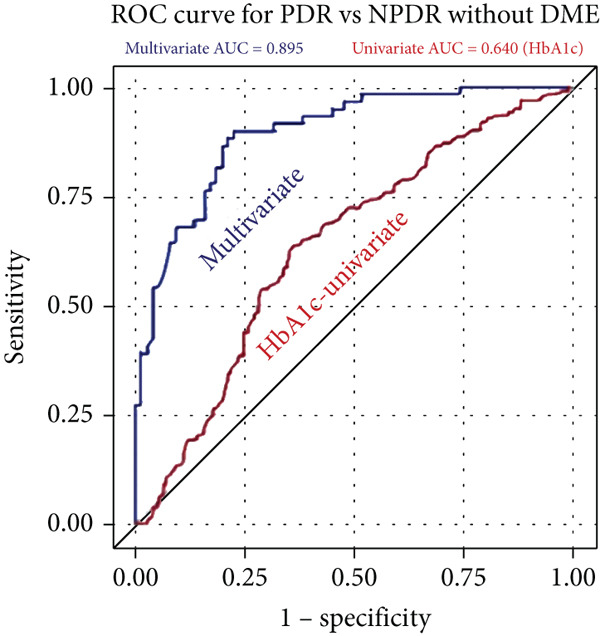
(a)

**Figure:**
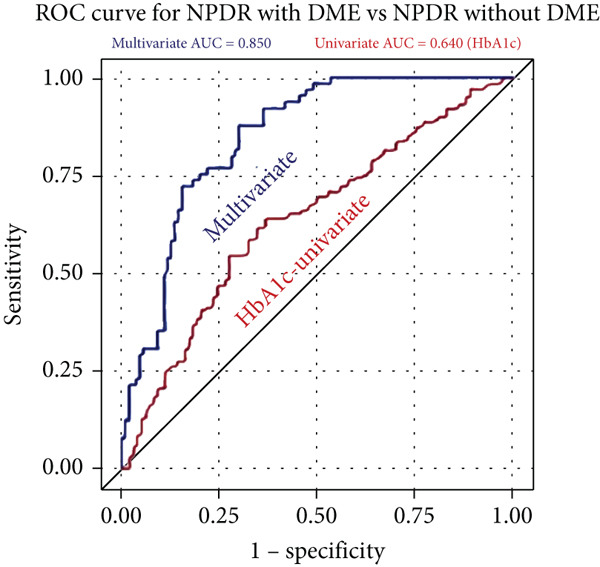
(b)

## 4. Discussion

In our study of a New Mexican population that consists of significant numbers of Hispanics and American Indians, we have identified several associative factors that may help in understanding the relationship of these factors and the risk of developing sight‐threatening complications like PDR and DME in patients with Type 2 diabetes. Our multivariate analysis revealed strong associative factors for developing PDR in this population, including duration of diabetes, Hispanic race, microalbuminuria and macroalbuminuria, and use of insulin and beta blockers. For DME development, the strongest associative factors included higher HbA1c level, microalbuminuria and macroalbuminuria, lower GFR, and an inverse relation with use of beta blockers. Our study found some significant new findings regarding the relationship between glycated hemoglobin and severity of retinopathy. The HbA1c level was not significantly associated with the development of PDR but was found to be significantly associated with DME. Also, the univariate HbA1c model in the ROC curve analysis showed its poor predictability in the development of both PDR and DME. As the Hispanics represent the fastest growing minority population in the United States, these new findings about the relationship between associative factors and DR severity may be important in further intervention programs that may be aimed at lowering the morbidity associated with PDR and DME.

In our multivariate logistic regression analysis, we could not find a significant association between HbA1c level and PDR presence. Our use of ROC curves demonstrated that solely using HbA1c was a “poor” marker of predictability for development of PDR and DME. Thus, glycemic exposure in our study does not reliably predict retinopathy status. The inconsistent association between HbA1c levels and PDR and DME development supports why many patients with adequate glycemic control develop severe retinopathy and why many patients with poor glycemic control have mild retinopathy. Although aggressive and chronic management of glycemic control is an important strategy in managing DR, the correlation has not always been so straightforward. A post hoc analysis of the DCCT showed that total glycemic exposure, including HbA1c, may only account for 11% of the risk of developing PDR [16]. Similarly, the Wisconsin Epidemiologic Study of Diabetic Retinopathy (WESDR) revealed that blood pressure and lipid control have been estimated to account for only 10% of the risk for developing disease [17]. Clinical evidence has also shown that some patients with diabetes have no clinical evidence of DR despite a duration of diabetes more significant than 50 years [18]. Two other population‐based studies, namely the LALES Study and Proyecto VER in the Latinos, failed to show a significant relationship between glycemic control and PDR development [5, 19]. A retrospective study in a US veteran population with Type 2 diabetes also showed that HbA1c levels had “little predictive value” for presence or severity of retinopathy at initial screenings [20].

There are likely factors other than glycemic exposure, possibly genetic factors, that may play a role in the development and progression of severe retinopathy. The severity of renal disease in long‐standing diabetes has often been associated with poorer functional outcomes in other organ systems as well. Chronic kidney disease, defined as increased severity of albuminuria and decreased GFR, was shown to be significantly associated with both PDR and DME in our subjects. Individuals with microalbuminuria are more likely to have retinopathy than those without microalbuminuria [21], suggesting that the severity of albuminuria may predict DR progression. DME has been associated with higher levels of UACR, the primary surrogate for albuminuria severity [22]. Abnormal renal profiles at baseline including high serum creatinine and low GFR were associated with PDR development in patients with Type 2 diabetes [23]. These results underscore a link between the microvascular complications of diabetes.

Race/ethnicity is associated in the development and progression of DR. However, these differences may more realistically be related to disparities in healthcare access, delayed diagnoses, existing comorbidities, and other social determinants of health. Studies have shown that Latino and Native American ancestry are associated with an increased severity of DR [7, 12]. Hispanics have an increased incidence and damage from intraretinal hemorrhage compared to African Americans despite similar prevalences of macular edema [10]. The exact culprit variants remain unknown, requiring further genomic analysis to identify specific genotypes and variations across ethnicities. Our multicenter Diabetic Retinopathy Genomics (DRGen) Study is currently examining the contribution of rare and common genetic variants in the development of DR [4]. The aim is to understand how these variants can contribute to clinical phenotypes, rates of progression, and responses to available therapies – ultimately allowing for the development of targeted therapy for specific individuals identified as being at risk. Nonetheless, it is critical to acknowledge that the differences observed among ethnic groups are highly complex and not solely determined by genetics. We recognize socioeconomic factors play a significant role in the health and disease status and are not measurable through genetic analyses or other factors investigated in this study.

There is well‐established clinical evidence that blood pressure control is associated with decreased severity of DR. Dysregulated retinal perfusion that results from hyperglycemia is accentuated with hypertension [24]. Additionally, hypertension has been associated with upregulation of vascular endothelial growth factor (VEGF) expression independent of hyperglycemia [25]. On the other hand, there is also a lack of evidence of any beneficial effect of intensive blood pressure control on any significant reduction in DR as shown in the ACCORD Study [3]. The choice of medication has been considered irrelevant so far as its effect is mediated through reduction of the blood pressure variable [26]. Though SBP was shown to be strongly associated with both PDR and DME development in our study, multivariate analysis suggests that the effect of beta‐blocker therapy on lowering SBP was primarily responsible for this. Thus, the observed protective association of beta blockers in our study may thus be confounded by the effect on hypertension. Beta blockers, as compared with other antihypertensive medication classes, theoretically have a negative effect on insulin sensitivity and release, but these relationships are poorly demonstrated in practice [5]. Insulin use was also shown to play a large role in the development of PDR in our population. Insulin is linked to angiogenesis, as insulin‐like growth factor 1 (IGF‐1) is expressed in increased amounts in patients with insulin use. IGF‐1 likely leads to neovascularization, the hallmark of PDR [27].

Our study had several limitations. First, this study is cross‐sectional in nature and does not support time‐sequential relationships; therefore, causality cannot be inferred. We refer to associative factors throughout the study, rather than risk or protective factors, due to this limitation [28]. Furthermore, although HbA1c was not significantly associated with PDR and only had a modest association with DME, this does not mean glycemic control is fully irrelevant. This study does not assess long‐term glycemic variability, which is shown to be a stronger predictor of DR than A1c alone [29]. In addition, laboratory values were collected retrospectively, and patient follow‐up was not done at regular intervals. Though many patients had ample results to calculate averages, some had missing values between follow‐up visits. We aimed to correct these variances by calculating averages over extended periods. We also performed statistical analyses on these values by categorizing them into strata to help account for the added variance this may have caused. Next, the variables of race/ethnicity and duration of diabetes likely have subjective variation, as patients were asked to self‐disclose their race/ethnicity and individually recollect their diabetes diagnosis date. Standardized retinal photographs were not available for all our patients for grading DR. Lastly, this study was designed as a cross‐sectional study with no defined temporality regarding risk factors and progression of DR.

In summary, the progression of DR is multifactorial and cannot be reliably predicted based on glycemic exposure alone, as defined by glycemic control and the duration of diabetes. Other factors, including the presence of diabetic nephropathy and race/ethnicity, appear to play a role in protecting or predisposing individuals to disease progression. This data may be useful for future investigations of risk factors for DR, which is the leading preventable cause of vision loss.

## Conflicts of Interest

The authors declare no conflicts of interest.

## Author Contributions

R.N.M.: Data curation, formal analysis, writing—original draft. L.M.: Data curation and formal analysis. G.A.: Data curation and formal analysis. A.P.C.: Data curation and formal analysis. F.M.: Writing—review and editing and formal analysis. C.Q.: Formal analysis, software, writing—review and editing, and supervision. A.D.: Funding acquisition, formal analysis, project administration, resources, writing—review and editing, supervision, writing—original draft, and conceptualization.

## Funding

This work was funded by the National Eye Institute (10.13039/100000053), NIH R01 028606‐A1.

## Data Availability

All data supporting the study results are available from the corresponding author, A.D., upon reasonable request.

## References

[1] Hainsworth D. P. , Bebu I. , Aiello L. P. , Sivitz W. , Gubitosi-Klug R. , Malone J. , White N. H. , Danis R. , Wallia A. , Gao X. , Barkmeier A. J. , Das A. , Patel S. , Gardner T. W. , and Lachin J. M. , Risk Factors for Retinopathy in Type 1 Diabetes: The DCCT/EDIC Study, Diabetes Care. (2019) 42, no. 5, 875–882, 10.2337/dc18-2308, 2-s2.0-85065073463, 30833368. 30833368 PMC6489114

[2] Kohner E. M. , Aldington S. J. , Stratton I. M. , Manley S. E. , Holman R. R. , Matthews D. R. , and Turner R. C. , United Kingdom Prospective Diabetes Study, 30, Archives of Ophthalmology. (1998) 116, no. 3, 297–303, 10.1001/archopht.116.3.297, 2-s2.0-0031916725. 9514482

[3] Chew E. Y. , Davis M. D. , Danis R. P. , Lovato J. F. , Perdue L. H. , Greven C. , Genuth S. , Goff D. C. , Leiter L. A. , Ismail-Beigi F. , Ambrosius W. T. , and Action to Control Cardiovascular Risk in Diabetes Eye Study Research Group , The Effects of Medical Management on the Progression of Diabetic Retinopathy in Persons With Type 2 Diabetes: The Action to Control Cardiovascular Risk in Diabetes (ACCORD) Eye Study, Ophthalmology. (2014) 121, no. 12, 2443–2451, 10.1016/j.ophtha.2014.07.019, 2-s2.0-84961289893, 25172198. 25172198 PMC4252767

[4] Cabrera A. P. , Mankad R. N. , Marek L. , Das R. , Rangasamy S. , Monickaraj F. , and Das A. , Genotypes and Phenotypes: A Search for Influential Genes in Diabetic Retinopathy, International Journal of Molecular Sciences. (2020) 21, no. 8, 10.3390/ijms21082712. PMC7215289 32295293

[5] West S. K. , Klein R. , Rodriguez J. , Muñoz B. , Broman A. T. , Sanchez R. , Snyder R. , and Proyecto V. E. R. , Diabetes and diabetic retinopathy in a Mexican-American population: Proyecto VER, Diabetes Care. (2001) 24, no. 7, 1204–1209, 10.2337/diacare.24.7.1204, 2-s2.0-0035408778. 11423503

[6] Varma R. , Paz S. H. , Azen S. P. , Klein R. , Globe D. , Torres M. , Shufelt C. , Preston-Martin S. , and Los Angeles Latino Eye Study Group , The Los Angeles Latino Eye Study design, Methods, and Baseline Data, Ophthalmology. (2004) 111, no. 6, 1121–1131, 10.1016/j.ophtha.2004.02.001, 2-s2.0-2942609145, 15177962. 15177962

[7] Gao X. , Gauderman W. J. , Marjoram P. , Torres M. , Chen Y. D. , Taylor K. D. , Rotter J. I. , and Varma R. , Native American Ancestry is Associated With Severe Diabetic Retinopathy in Latinos, Investigative Ophthalmology & Visual Science. (2014) 55, no. 9, 6041–6045, 10.1167/iovs.14-15044, 2-s2.0-84908110235, 25146985. 25146985 PMC4176415

[8] New Mexico Data Focus Hispanic or Latino ethnicity , 2024, https://www.dws.state.nm.us/Portals/0/DM/LMI/NM_Data_Focus_Hispanic_Latino_Ethnicity.pdf.

[9] Nations, Pueblos and Tribes , NM Indian Affairs Department, 2023, https://www.lad.nm.gov.

[10] Chen J. L. , Luviano D. M. , Chen J. C. , Yu F. , and Sarraf D. , Comparison of Diabetic Retinopathy Phenotype Between Latinos and Blacks, Journal of Diabetes and its Complications. (2009) 23, no. 6, 371–375, 10.1016/j.jdiacomp.2008.05.001, 2-s2.0-70350490519. 18599323

[11] Gillow J. T. , Gibson J. M. , and Dodson P. M. , Hypertension and Diabetic Retinopathy--What′s the Story?, The British Journal of Ophthalmology. (1999) 83, no. 9, 1083–1087, 10.1136/bjo.83.9.1083, 2-s2.0-0032845265. 10460781 PMC1723193

[12] Mora N. , Kempen J. H. , and Sobrin L. , Diabetic Retinopathy in Hispanics: A Perspective on Disease Burden, American Journal of Ophthalmology. (2018) 196, xviii–xxiv, 10.1016/j.ajo.2018.08.021, 2-s2.0-85054165334, 30138600. 30138600

[13] Levey A. S. , Stevens L. A. , Schmid C. H. , Zhang Y. L. , Castro A. F.3rd, Feldman H. I. , Kusek J. W. , Eggers P. , Van Lente F. , Greene T. , and Coresh J. , A new equation to estimate glomerular filtration rate, Annals of Internal Medicine. (2009) 150, no. 9, 604–612, 10.7326/0003-4819-150-9-200905050-00006, 2-s2.0-65649142017, 19414839. 19414839 PMC2763564

[14] Diabetic Retinopathy Clinical Research Network; Wells JA, Glassman AR, Ayala AR, Jampol LM, Aiello LP, Antoszyk AN, Arnold-Bush B, Baker CW, Bressler NM, Browning DJ, Elman MJ, Ferris FL, Friedman SM, Melia M, Pieramici DJ, Sun JK, Beck RW , Aflibercept, Bevacizumab, or Ranibizumab for Diabetic Macular Edema, New England Journal of MedicineMar. (2015) 372, no. 13, 1193–1203, 10.1056/NEJMoa1414264, 2-s2.0-84925423332. PMC4422053 25692915

[15] Mandrekar J. N. , Receiver Operating Characteristic Curve in Diagnostic Test Assessment, Journal of Thoracic Oncology. (2010) 5, no. 9, 1315–1316, 10.1097/JTO.0b013e3181ec173d, 2-s2.0-77956244594. 20736804

[16] Lachin J. M. , Genuth S. , Nathan D. M. , Zinman B. , Rutledge B. N. , and DCCT/EDIC Research Group , Effect of Glycemic Exposure on the Risk of Microvascular Complications in the Diabetes Control and Complications Trial--Revisited, Diabetes. (2008) 57, no. 4, 995–1001, 10.2337/db07-1618, 2-s2.0-42449121829, 18223010. 18223010

[17] Klein R. , Klein B. E. , Moss S. E. , and Cruickshanks K. J. , The Wisconsin Epidemiologic Study of Diabetic Retinopathy: XVII. The 14-Year Incidence and Progression of Diabetic Retinopathy and Associated Risk Factors in Type 1 Diabetes, Ophthalmology. (1998) 105, no. 10, 1801–1815, 10.1016/S0161-6420(98)91020-X, 2-s2.0-0031793875, 9787347. 9787347

[18] Sun J. K. , Keenan H. A. , Cavallerano J. D. , Asztalos B. F. , Schaefer E. J. , Sell D. R. , Strauch C. M. , Monnier V. M. , Doria A. , Aiello L. P. , and King G. L. , Protection From Retinopathy and Other Complications in Patients With Type 1 Diabetes of Extreme Duration: The Joslin 50-Year Medalist Study, Diabetes Care. (2011) 34, no. 4, 968–974, 10.2337/dc10-1675, 2-s2.0-79956213781, 21447665. 21447665 PMC3064059

[19] Varma R, Macias GL, Torres M, Klein R, Peña FY, Azen SP; Los Angeles Latino Eye Study Group , Biologic Risk Factors Associated With Diabetic Retinopathy: The Los Angeles Latino Eye Study, Ophthalmology. (2007) 114, no. 7, 1332–1340, 10.1016/j.ophtha.2006.10.023, 2-s2.0-34347223266. 17306879

[20] Maa A. Y. and Sullivan B. R. , Relationship of Hemoglobin A1C With the Presence and Severity of Retinopathy Upon Initial Screening of Type II Diabetes Mellitus, American Journal of Ophthalmology. (2007) 144, no. 3, 456–457, 10.1016/j.ajo.2007.04.008, 2-s2.0-34548223050, 17765430. 17765430

[21] Cruickshanks K. J. , Ritter L. L. , Klein R. , and Moss S. E. , The Association of Microalbuminuria With Diabetic Retinopathy, Ophthalmology. (1993) 100, no. 6, 862–867, 10.1016/s0161-6420(93)31562-9, 2-s2.0-0027229167. 8510898

[22] Zhuang X. , Cao D. , Yang D. , Zeng Y. , Yu H. , Wang J. , Kuang J. , Xie J. , Zhang S. , and Zhang L. , Association of Diabetic Retinopathy and Diabetic Macular Oedema With Renal Function in Southern Chinese Patients With Type 2 Diabetes Mellitus: A Single-Centre Observational Study, BMJ Open. (2019) 9, no. 9, e031194, 10.1136/bmjopen-2019-031194, 2-s2.0-85071896302, 31494622. PMC6731866 31494622

[23] Hsieh Y. T. , Tsai M. J. , Tu S. T. , and Hsieh M. C. , Association of Abnormal Renal Profiles and Proliferative Diabetic Retinopathy and Diabetic Macular Edema in an Asian Population With Type 2 Diabetes, JAMA Ophthalmol. (2018) 136, no. 1, 68–74, 10.1001/jamaophthalmol.2017.5202, 2-s2.0-85041129774, 29167896. 29167896 PMC5833599

[24] Srivastava B. K. and Rema M. , Does Hypertension Play a Role in Diabetic Retinopathy?, The Journal of the Association of Physicians of India. (2005) 53, 803–808, 16334627. 16334627

[25] Do D. V. , Wang X. , Vedula S. S. , Marrone M. , Sleilati G. , Hawkins B. S. , and Frank R. N. , Blood Pressure Control for Diabetic Retinopathy, São Paulo Medical Journal. (2015) 133, no. 3, 278–279, 10.1590/1516-3180.20151333T1, 2-s2.0-84937432033, 26176931. 26176931 PMC10876377

[26] Wicklmayr M. , Rett K. , Dietze G. , and Mehnert H. , Effects of Beta-Blocking Agents on Insulin Secretion and Glucose Disposal, Hormone and Metabolic Research Supplement. (1990) 22, 29–33. 1975244

[27] Ruberte J. , Ayuso E. , Navarro M. , Carretero A. , Nacher V. , Haurigot V. , George M. , Llombart C. , Casellas A. , Costa C. , Bosch A. , and Bosch F. , Increased Ocular Levels of IGF-1 in Transgenic Mice Lead to Diabetes-Like Eye Disease, The Journal of Clinical Investigation. (2004) 113, no. 8, 1149–1157, 10.1172/JCI19478, 2-s2.0-11144354304, 15085194. 15085194 PMC385397

[28] Hsieh Y. T. and Hsieh M. C. , Time-Sequential Correlations Between Diabetic Kidney Disease and Diabetic Retinopathy in Type 2 Diabetes - an 8-Year Prospective Cohort Study, Acta Ophthalmologica. (2021) 99, no. 1, e1–e6, 10.1111/aos.14487, 32567151. 32567151

[29] Hsieh Y. T. and Hsieh M. C. , Fasting Plasma Glucose Variability is an Independent Risk Factor for Diabetic Retinopathy and Diabetic Macular Oedema in Type 2 Diabetes: An 8-Year Prospective Cohort Study, Clinical & Experimental Ophthalmology. (2020) 48, no. 4, 470–476, 10.1111/ceo.13728, 32065699. 32065699

